# Biological Society of New York

**Published:** 1853-10

**Authors:** 


### ECLECTIC DEPARTMENT,

### AND SPIRIT OF THE MEDICAL PERIODICAL PRESS.

*Biological Society of New York.* Extracts from the minutes, by JNO. C. DALTON, Jr., M. D., Secretary.

Meeting of July 1st, 1853.—Dr. Dalton exhibited some specimens of fish and salamanders taken from the interior of Howe's cave, in Schoharie county, N. Y. Several months ago, having occasion to dissect some specimens of *Ambiyopsis spelœus* from the Mammoth Cave in Kentucky, and of *Proteus anguinus* from the Grotto of Carniola, he had become particularly interested in the following question, viz:

What relation exists between the imperfect development of the organs of sight in these animals, and the fact that they live only in darkness? Is the blindness a case of degeneration, or atrophy ftom disuse, owing to the animals never having access to light; or is it simply an original specific characteristic, like size or shape, or external markings of the integument.

The Mammoth Cave and the Carniola grottoes are the only localities at present known to contain these peculiar species of animals. In the Proteus, the eyes are destitute of lids, and lie beneath the common integument, which is continued over them unchanged. The whole integument, however, in this animal is so transparent that usually the eye can be seen through it as a small blackish point. In an individual not quite nine inches in length, the eyeball measured one-fiftieth of an inch in diameter, and consisted of a sclerotic, with blackish pigment, a globular crystalline lens, an imperfect iris, and a cornea. The fish from the Mammoth Cave have been said to be entirely without eyes; but this is not the case. In an individual four and seven-eighths inches long, the eyes were to be seen as minute globular sacs, containing blackish pigment, deeply embedded in the adipose tissue of the orbit, and measuring a little less than one seventy-eighth of an inch in diameter. It is, then, not an absence, but an imperfect development of the organs of vision which characterizes these animals. In this respect the Proteus is resembled, more or less, by other species of ichthyoid reptiles, found in other localities. In the *Lepidosiren paradoxa*, examined and described by Bischoff, the eyeballs were "hardly a line in diameter," though one of the animals was over three feet in length. The eyelids were also wanting, and the common integument continuous over the eye, only more transparent here than elsewhere. The small size of the eyeball and the absence of lids, are also characteristic of *Menobranchus*, and other ichthyoid reptiles of this country, which live in the open waters, and have as much access to light as any other aquatic animal. The imperfection in the visual organs of Proteus must, then, be considered, to a great extent, as characteristic of a family, and not altogether dependent on the peculiar circumstances surrounding the species.

It is noticeable, that the suppression of visual organs in subterranean species is *complete* only in Invertebrata, (Crustaceans and Arachnida, existing both in the Mammoth Cave and Carniola grottoes, in which no trace of eyes has yet been found;) that it is nearly complete in the lowest class of vertebrata, fish; and decidedly less so in that next above, viz., reptiles. The mammalians, (rats, &c.,) taken from the Mammoth Cave are said to show no deficiency in the development of the eyes, nor any of that absence of color which is so striking in the blind fish and in the Proteus.

In the month of March, last, thinking it possible that animals more or less blind, might exist in other subterranean situations than those already examined, Dr. D. made arrangements to procure from Howe's Cave all the fish and reptiles that might be taken there during the summer. The cavern is situated in the town of Cobleskill, some thirty miles south-west from Albany. It penetrates the side of a limestone hill in a nearly horizontal direction. The passage soon descends abruptly twelve or fifteen feet, after which it continues again a horizontal course. Not quite half a mile from the entrance is a sheet of water, of which the origin and outlet are concealed. During the summer months, it may be crossed by boats, and the cave explored for some six miles beyond. The proprietor, Mr. Howe, states that he has himself penetrated eleven miles. In the summer of 1849, wishing to stock his pond, he put into the waters of the cave specimens of trout, dace, suckers, and shiners, about a dozen of each; but has never introduced any reptiles or any other species of fish.

The specimens recently obtained from the cave, and sent here for examination, consist of four fish and two reptiles. The fish are specimens of suckers and dace, (Catostomus and Leuciscus,) and are, in all probability, the progeny of those introduced four years ago by Mr. Howe. They are of small size, the dace measuring four to four and a quarter inches in length, the suckers three and a half to four. Otherwise they are not remarkable.

The reptiles are both Salamanders. One was found at a distance of sixty yards, the other about three miles from the mouth of the cavern. One of them corresponds in all essential particulars with *Salamandra symmetrica*, the other with *S. subviolacea*, (De Kay.) They are, respectively, 3.2 and 4.4 inches in length. The yellow color of the dorsal and lateral spots of the *S. subviolacea*, has been, apparently, removed by the action of the spirit in which the specimens have been kept—the spots being at present of a dead white; and the red spots of *S. symmetrica* are now becoming decolorized from the same cause. Otherwise the animals present no peculiarity. The eyes and eyelids are perfectly well developed in each.

The information derived from these specimens, though very imperfect, is of value, so far as it goes. They show that species of reptiles, identical with those living in the open air, may exist in subterranean recesses with complete absence of light, and yet suffer no modification of the visual organs. Still, it is uncertain how long these animals had inhabited the cave; and further explorations of this and similar localities, may yet discover new and peculiar species, from which more definite information can be obtained.

Dr. Gilman thought the question of the possible modification of specific characters, by the influence of external circumstances, one of extreme interest. He remarked that, though the imperfection of the eyes in *Proteus Anguinus* was to a certain extent characteristic of the ichthyoid family of reptiles, and not confined to that particular species, yet this would not apply to the blind fish, or the crustaceans of the Mammoth Cave, in which the imperfection was much more strongly marked. He hoped that the exploration of the various caverns in the United States would be continued, and their zoology thoroughly examined with reference to this point.—*N. Y. Med. Times.*

